# Innovative models to explore hepatic involvement in Prader-Willi syndrome

**DOI:** 10.3389/fendo.2026.1794669

**Published:** 2026-06-24

**Authors:** Romar Guintu Dabban, Graziano Grugni, Adele Bondesan, Benedetta Blarasin, Claudio Tiribelli, Cristina Bellarosa, Alessandro Sartorio

**Affiliations:** 1 Fondazione Italiana Fegato (Italian Liver Foundation), Innovative Models Unit, Trieste, Italy; 2 Department of Life Sciences, University of Trieste, Trieste, Italy; 3 Philippine Council for Health Research and Development, Department of Science and Technology, Taguig, Metro Manila, Philippines; 4 Istituto Auxologico Italiano, IRCCS (Istituto di Ricovero e Cura a Carattere Scientifico), Experimental Laboratory for Auxo-Endocrinological Research, Piancavallo-Verbani, Italy

**Keywords:** hepatocyte-like cells, iPSCs, lipid metabolism, liver organoids, Prader-Willi syndrome

## Abstract

Prader-Willi syndrome (PWS; MIM# 176270) is a rare neurodevelopmental disorder characterized by clinical manifestations across multiple body systems. PWS represents an atypical form of obesity-associated metabolic disease in which extreme adiposity coexists with a comparatively attenuated risk of insulin resistance and hepatic complications. The genetic basis of PWS leads to impaired lipid storage and oxidation capacity in adipocytes, with downstream consequences for hepatic lipid burden. Systemic lipidomic and metabolomic profiling further supports the existence of a distinct metabolic signature in PWS, showing consistent qualitative alterations in circulating phospholipids that may, in turn, influence hepatic lipid export and the risk of steatosis. Hepatic involvement in PWS seems to be shaped by intrinsic alterations in lipid handling and endocrine signaling rather than by adiposity. Finally, metabolic dysfunction-associated steatotic liver disease (MASLD) remains less prevalent in PWS. Among the currently available models derived from induced pluripotent stem cells (iPSCs), hepatocyte-like cells (HLCs) and iPSC-derived organoids, have emerged as valuable tools for investigating rare genetic liver disorders and as powerful strategies to dissect tissue-specific mechanisms underlying PWS phenotype, because they bridge the gap between molecular epigenetic mechanisms and organism-level metabolic phenotypes in PWS. Their ability to capture patient-specific epigenetic regulation and tissue-specific metabolic dysfunction positions them as indispensable tools for advancing both mechanistic understanding and therapeutic development in PWS.

## 1 Introduction

Prader-Willi syndrome (PWS; MIM# 176270) is a rare neurodevelopmental disorder characterized by clinical manifestations across multiple body systems (1). PWS is recognized as the most common syndromic cause of life-threatening obesity and affects males and females equally. It occurs in approximately 1 in 21,000 live births (2) and is reported at 10.7% among infants with hypotonia (3). The syndrome is caused by the failure to express a cluster of paternally expressed genes in the PWS region of chromosome 15 (15q11.2-q13).

### 1.1 Genetics of PWS

The main genetic mechanisms responsible for PWS are: (1) paternal 15q11.2-q13 deletion (del15) (60-70% of cases); (2) maternal uniparental disomy for chromosome 15 (UPD15), in which both copies of chromosome 15 are derived from the mother (25-35% of cases); (3) imprinting center defect (IC) and other chromosome 15 alterations (1-4%) (4).

There are two types of deletion on the paternal chromosome 15: type I and type II. Moreover, approximately 8% of individuals with del15 show an unusual or atypical deletion size (5). The type I deletion is larger and extends from the chromosome 15 proximal breakpoint 1 (BP1) to the distal breakpoint BP3. In contrast, the type II deletion is smaller and involves chromosome 15 proximal breakpoint BP2, located ~500 kb distal to BP1, and distal breakpoint BP3 (4).

The PWS critical region lies between BP2 and BP3 and harbors a cluster of imprinted genes that are crucial for appetite, sleep, neurodevelopment, metabolism, and hormonal regulation. These genes include makorin ring finger protein 3 (*MKRN3*), MAGE family member L2 (*MAGEL2*), necdin, MAGE family member (*NDN*), small nuclear ribonucleoprotein polypeptide N (*SNURF-SNRPN*), small nucleolar RNA, C/D box 116 (*SNORD116*), and nuclear pore associated protein 1 (*C15orf2/NPAP1*), but none of them accounts for the complete PWS phenotype (6). Additionally, the genotype-phenotype correlation in PWS remains unclear, as no phenotypic feature is known to be exclusively associated with any of the three main molecular mechanisms (7, 8).

### 1.2 Clinical and metabolic features of PWS

The phenotype of individuals with PWS varies significantly across the lifespan, becoming more evident in adulthood (9). Furthermore, the clinical picture of affected patients can vary among individuals, both in severity and in specific symptoms.

Clinical manifestations of PWS (**Table 1**) include severe neonatal hypotonia and feeding difficulties in early infancy leading to failure to thrive, followed by progressive hyperphagia with early childhood-onset obesity and associated comorbidities, unless feeding is promptly restricted. Other manifestations include learning disabilities, behavioral disorders and increased risk of psychosis, dysmorphic features such as peculiar facial appearance, small hands and feet, scoliosis and/or kyphosis (10). Several endocrinopathies are described in PWS, including hypogonadotropic and hypergonadotropic hypogonadism, precocious puberty, growth hormone (GH)/insulin-like growth factor-I (IGF-I) axis dysfunction, hypothyroidism, impaired oxytocin system, ghrelin dysfunction, premature adrenarche and, rarely, central adrenal insufficiency (11, 12). Additionally, the syndrome is characterized by central sleep apnea (commonly observed up to 2 years of age), epilepsy, esotropia, decreased vomiting, high pain threshold and temperature abnormalities. Impaired hypothalamic function is currently thought to underlie many signs and symptoms of this multifaceted phenotype (13).

**Table 1:** Clinical problems associated with Prader-Willi syndrome.

Obesity and its metabolic complications	Hyperphagia and early-childhood onset of severe obesity (if uncontrolled), IFG, IGT, T2DM, dyslipidemia, high uric acid, hypovitaminosis D, metabolic syndrome
Endocrine alterations	hypogonadism, precocious puberty, GH/IGF-I axis dysfunction, hypothyroidism, premature adrenarche, CAI, altered oxytocin system, hyperghrelinemia
Cardiovascular diseases	Heart failure, dilated cardiomyopathy, ischemic heart disease, cardiac arrhythmias, high blood pressure, venous thrombosis, and lymphedema in the lower extremities
Respiratory abnormalities	Central sleep apnea, obstructive sleep apnea, hypersomnia, and altered sleep architecture
Skeletal problems	Scoliosis and/or kyphosis, small hands and feet, straight ulnar borders, hip dysplasia, distinctive facial features, osteopenia/osteoporosis
Muscular issues	Muscular hypotonia (responsible for decreased fetal movements, initial poor suck with failure to thrive, and increased choking risk), decreased lean mass, and joint hypermobility
Gastrointestinal alterations	Thick viscous saliva, gastric emptying deficiency and risk of gastric necrosis, constipation, MASLD, and cholelithiasis
Brain	Intellectual and learning disability, obsessive-compulsive behavior, self-mutilation (skin picking, rectal picking, hair pulling), psychiatric illness, speech articulation defects, early dementia (debated)
Hypothalamic dysfunction	Thermoregulatory disorders, high pain threshold, decreased vomiting, and hydroelectrolytic disorders
Ocular problems	Esotropia, hypermetropia, myopia
Miscellaneous	Epilepsy, nocturnal and/or daytime urinary incontinence, fecal incontinence, unusual skill with jigsaw puzzles, hypopigmentation of hair, eyes, and skin (compared with family), dental and orthodontic issues

IFG, impaired fasting glucose; IGT, impaired glucose tolerance; T2DM, type 2 diabetes mellitus; GH, growth hormone; IGF-I, insulin-like growth factor-I; CAI, central adrenal insufficiency; MASLD, metabolic dysfunction-associated steatotic liver disease. GH/IGF-I axis, growth hormone/insulin-like growth factor-I axis; HDL, high-density lipoprotein; IFG, impaired fasting glucose; IGT, impaired glucose tolerance.

In recent years, early diagnosis and changes in medical and behavioral supervision appear to have significantly increased the life expectancy of patients with PWS (14). Despite these advances, the mortality rate of subjects with PWS remains considerably higher than in the general population (15). Morbidity and mortality are mainly related to the presence of obesity and its complications (type 2 diabetes mellitus (T2DM), heart diseases, arterial hypertension and obstructive sleep apnea) (16, 17), particularly in adult age (18).

In this context, obesity in subjects with PWS presents peculiar phenotypic features that are unusual in non-syndromic obesity. Individuals with PWS have higher fat mass and significantly lower fat-free mass than those with simple obesity at the same body mass index (BMI) level throughout their lifespans (19). Excessive fat mass in PWS typically affects the trunk and the proximal extremities of the limbs. Unlike simple obesity, patients with PWS show less visceral adiposity and a lower trunk-to-appendicular fat mass ratio (20; G. 21–23). According to these results, patients with PWS have been reported to have greater insulin sensitivity and a healthier lipid profile than subjects with simple obesity (18, 24). However, data on fat distribution and insulin secretion are still conflicting. A similar proportion of visceral fat was detected in obese subjects with PWS compared to BMI-matched controls (25). Furthermore, other authors observed that insulin levels and insulin resistance were similar in obese patients with PWS and obese controls at all ages (26). Accordingly, obesity in PWS shows a prevalence of metabolic syndrome (MetS) similar to that of controls with simple obesity, both in children, adolescents and adult subjects (G. 27, 28). These discrepancies could be related to the different clinical characteristics of the study groups, such as age, degree of obesity, and presence/absence of therapy with GH and/or sex steroids.

Metabolic complications usually associated with obesity in PWS include altered glucose metabolism, dyslipidemia, hyperuricemia, hypovitaminosis D, and metabolic dysfunction-associated steatotic liver disease (MASLD). Impaired glucose metabolism is common in PWS and affects 10-25% of patients, particularly in obese and adult subjects (26). Considering the specific subtypes of altered glucose homeostasis, a multicenter study of 274 Italian patients with PWS found that 0.7% had impaired fasting glucose, 10.2% had impaired glucose tolerance, and 13.5% had T2DM (29). Few data are currently available on the prevalence of dyslipidemia in either children or adults with PWS (15, 16, 18, 28). In this context, it has been recently reported that 40% of patients with PWS had an altered lipid profile, single or combined (11). Like other metabolic alterations, hyperuricemia is influenced by age and obesity, with a higher frequency in adults, although its prevalence in PWS appears lower than in obese controls (30).

## 2 Hepatic diseases and metabolic dysregulation in PWS

PWS represents an atypical form of obesity-associated metabolic disease in which extreme adiposity coexists with a comparatively attenuated risk of insulin resistance and hepatic complications (10, 31; Y. 23, 32–34). Unlike nonsyndromic obesity, where excess caloric intake and progressive insulin resistance are central drivers of metabolic disease, PWS is characterized by intrinsic abnormalities in adipose tissue development, lipid partitioning, and endocrine regulation (35–38). These syndrome-specific features shape hepatic lipid exposure and influence susceptibility to MASLD. This section critically examines current evidence linking altered lipid handling, metabolomic signatures, and hepatic disease risk in PWS, with emphasis on mechanisms relevant to liver metabolism.

### 2.1 Lipid handling and metabolomic signatures in PWS

Metabolic alterations in PWS appear early in life and cannot be fully explained as secondary consequences of obesity alone. Even prior to overt weight gain, children with PWS exhibit abnormalities in white adipose tissue (WAT) structure and function that are likely to influence systemic lipid flux and hepatic lipid delivery (39, 40). Histological and cellular studies indicate that WAT expansion in PWS is characterized by altered cellular composition and low-grade immune cell infiltration, consistent with early inflammatory remodeling. Such changes are known to compromise adipose tissue lipid-buffering capacity and promote ectopic lipid deposition, particularly in the liver (35).

At the cellular level, adipose progenitors from PWS display impaired adipogenic differentiation. Reduced lipid accumulation, diminished triglyceride storage, and suppression of core adipogenic regulators, including peroxisome proliferator-activated receptor gamma (PPARG) and CCAAT enhancer binding protein alpha (CEBPA), have been reported across multiple experimental systems (35). Rather than supporting metabolically protective adipocyte hyperplasia, adipose expansion in PWS appears biased toward hypertrophy, a pattern strongly associated with lipid spillover and hepatic steatosis in nonsyndromic obesity (35). Impaired adipokine secretion from mature adipocytes further exacerbates this phenotype by weakening systemic metabolic coordination.

Defects in adipocyte lineage plasticity extend to beige adipogenesis. Beige adipocytes contribute to lipid oxidation and adaptive thermogenesis, thereby limiting lipid overload in peripheral tissues. In PWS-derived adipose cells, expression of browning regulators and mitochondrial genes is consistently reduced, accompanied by lower mitochondrial content and oxidative capacity (35). Functional assays demonstrate impaired mitochondrial respiration and reduced metabolic flexibility, favoring lipid redistribution toward the liver despite relatively preserved insulin sensitivity at the whole-body level (35).

Transcriptomic profiling of adipose-derived mesenchymal stem cells reinforces this model. Pro-adipogenic and insulin-responsive pathways are broadly suppressed, while anti-adipogenic and proliferative signals are enriched (36, 37). Increased proliferative capacity coupled with impaired terminal differentiation results in an expanded pool of immature adipose cells that are incapable of efficient lipid storage. This imbalance likely increases circulating free fatty acids and enhances hepatic lipid uptake (35).

Genetic studies implicate loss of SNORD116, a key component of the PWS critical region, as a driver of adipogenic dysfunction. Experimental disruption of SNORD116 recapitulates key features of the PWS adipocyte phenotype, including reduced PPARG expression and altered splicing of genes involved in adipocyte fate determination, such as PR/SET domain 16 (PRDM16) (35). These findings provide a direct mechanistic link between the genetic basis of PWS and impaired lipid storage and oxidation capacity, with downstream consequences for hepatic lipid burden.

Systemic lipidomic and metabolomic profiling further supports the existence of a distinct metabolic signature in PWS. Consistent alterations in circulating phospholipids, particularly phosphatidylcholines (PCs) and lysophosphatidylcholines (LPCs), have been reported (41, 42). PCs are essential for hepatic triglyceride packaging and very-low-density lipoprotein (VLDL) secretion; qualitative changes in PCs composition may therefore influence hepatic lipid export and steatosis risk (van der 43, 44). Importantly, these changes appear selective rather than global, suggesting regulated remodeling rather than generalized lipid dysregulation.

Alterations in LPC species, which function as bioactive mediators of inflammation and glucose metabolism, further distinguish PWS from nonsyndromic obesity (41). Species-specific LPC patterns may reflect differential regulation of enzymes linking PC metabolism to high-density lipoprotein (HDL) function, consistent with the relatively higher HDL cholesterol levels observed in PWS (41). Together, these lipidomic features may contribute to a less atherogenic systemic profile while still permitting hepatic lipid accumulation under specific conditions.

Triglyceride metabolism highlights the intermediate metabolic phenotype of PWS. Although circulating triglyceride levels are elevated relative to lean controls, they are generally lower than in BMI-matched nonsyndromic obesity, despite comparable fat mass (42). Moreover, selective depletion of specific triglyceride species suggests qualitative differences that may influence lipotoxicity and hepatic lipid partitioning.

Finally, acylcarnitine profiling provides insight into mitochondrial fatty acid oxidation. Elevated levels of medium- and short-chain acylcarnitines indicate incomplete β-oxidation beyond mitochondrial entry steps, consistent with broader mitochondrial dysfunction (41). Such defects may compromise hepatic energy metabolism and further modulate lipid handling.

### 2.2 Molecular features and biomarkers of hepatic steatosis in PWS

Hepatic steatosis is a common manifestation of metabolic dysfunction in obesity, yet its presentation in PWS is quantitatively and qualitatively distinct. Despite severe adiposity, individuals with PWS typically exhibit reduced visceral fat, lower fasting insulin levels, and relatively preserved insulin sensitivity (31; Y. 23, 32, 34, 45). These features suggest that hepatic involvement in PWS is shaped by intrinsic alterations in lipid handling and endocrine signaling rather than by adiposity.

Efforts to identify non-invasive biomarkers of hepatic steatosis have become increasingly relevant, particularly in PWS, where conventional clinical predictors may underestimate liver involvement. Circulating adipokines and lipid-binding proteins, including fatty acid binding protein 4 (FABP4) (M. 46–49), retinol binding protein 4 (RBP4) (50), and adiponectin (51, 52), have been linked to hepatic lipid accumulation and metabolic dysfunction. Proteomics-based approaches have expanded this landscape, identifying composite protein signatures associated with steatosis severity and fibrosis risk (53).

Among emerging candidates, angiopoietin-like 8 (ANGPTL8) has attracted particular interest. ANGPTL8 is predominantly expressed in the liver, localizes to lipid droplets, and regulates triglyceride trafficking through inhibition of lipoprotein lipase (54). In humans, circulating ANGPTL8 levels positively correlate with triglycerides and VLDL levels, positioning it at the interface of hepatic and systemic lipid metabolism (55).

In PWS, ANGPTL8 levels are consistently lower than in nonsyndromic obesity, paralleling lower triglycerides and higher HDL cholesterol (55). Reduced ANGPTL8 may facilitate triglyceride clearance and contribute to a relatively favorable lipid profile. Notably, ANGPTL8 shows weak associations with insulin resistance and glucose homeostasis in PWS, aligning with growing evidence that its role in human β-cell function is limited (56).

Proteomic studies further identify hepatic enzymes, including carboxylesterase 1 (CES1), fructose-bisphosphatase 1 (FBP1), and quinoid dihydropteridine reductase (QDPR), as markers associated with steatosis in PWS (57). CES1, in particular, is implicated in hepatic triglyceride storage and VLDL metabolism wherein experimental loss of CES1 reduces hepatic lipid accumulation, suggesting a modulatory role in steatosis susceptibility (58). Immune-associated proteins, including sialic acid binding Ig like lectin 7 (SIGLEC7) and dipeptidyl peptidase 7 (DPP7), correlate with disease severity and may reflect macrophage activation and fibrotic remodeling, implicating immune-metabolic crosstalk in progressive liver involvement (59–61).

### 2.3 MASLD in PWS

Despite severe and early-onset obesity, MASLD prevalence and severity are consistently lower in individuals with PWS than in nonsyndromic obesity (55, 62). This paradox highlights the presence of syndrome-specific modifiers of hepatic disease risk. Altered body composition, characterized by high fat mass but reduced lean mass, coexists with relative hypoinsulinemia and preserved insulin sensitivity (22, 45). When matched for percent body fat rather than BMI, insulin sensitivity is similar between PWS and controls, yet MASLD remains less prevalent in PWS, indicating that insulin sensitivity alone does not explain hepatic protection (63).

Evidence for a milder hepatic phenotype extends across age groups, with reduced MASLD prevalence reported in adult women and lower steatosis grades observed in pediatric cohorts (62–64). Furthermore, hepatic phenotypes do not appear to be strongly influenced by the different PWS genetic subtypes, as similar rates of MASLD have been reported in individuals with paternal deletions and those with maternal uniparental disomy; indicating that the relative protection from severe hepatic disease may be a shared feature of the syndrome rather than a subtype-specific phenomenon (65).

Endocrine factors, particularly GH deficiency and replacement therapy, further modulate MASLD risk. GH influences hepatic lipid oxidation and VLDL secretion, and replacement therapy improves body composition and reduces liver fat in GH-deficient states (66). While MASLD associations persist after adjusting for GH exposure, its contributory role warrants careful longitudinal evaluation (63).

## 3 Innovative models in hepatic disease research and their relevance to PWS

Understanding the mechanisms underlying hepatic involvement in PWS remains challenging because clinical observations often reveal a paradoxical phenotype characterized by severe obesity but relatively lower susceptibility to MASLD compared with individuals with nonsyndromic obesity. Experimental models capable of recapitulating human liver biology are therefore essential to investigate the cellular and molecular basis of this phenomenon. Traditional models, such as primary human hepatocytes (67), immortalized cell lines (68), and animal models (69), have contributed substantially to liver research (van der 70). However, each presents important limitations related to availability, genetic heterogeneity, species-specific differences, or loss of hepatic function in culture (71). In recent years, induced pluripotent stem cell-derived hepatocyte-like cells (iPSC-derived HLCs) and liver organoids have emerged as physiologically relevant human models of liver disease mechanisms while preserving patient-specific genetic backgrounds. These platforms are particularly attractive for studying PWS, where hepatic manifestations are likely influenced by complex interactions between genetics, epigenetics, adipose tissue dysfunction, and systemic metabolic regulation.

### 3.1 Hepatocyte-like cells from iPSCs

Among iPSC-derived hepatic models, HLCs have become widely used for studying inherited and metabolic liver diseases. Through stepwise differentiation that recapitulates embryonic liver development, iPSCs can be directed through definitive endoderm and hepatic progenitor stages before acquiring key hepatocyte characteristics, including albumin secretion, glycogen storage, drug metabolism, and lipid handling (72). Importantly, iPSCs can be generated from readily accessible patient tissues, including skin fibroblasts (73), blood cells (74, 75), and urine-derived epithelial cells (76), providing a renewable source of genetically matched HLCs for disease modeling.

HLCs have already been successfully applied to investigate numerous genetic liver disorders, including α1-antitrypsin deficiency (77), Wilson’s disease (78), progressive familial intrahepatic cholestasis (79), Pompe disease (80), and other inherited metabolic disorders (81). Their ability to reproduce disease-specific phenotypes while maintaining patient genetic backgrounds makes them particularly suitable for studying disorders in which cellular metabolism is intrinsically altered.

Although stem-cell derived adipocyte models have already provided important insights into lipid metabolism in PWS, the hepatic compartment remains largely unexplored (82). Extending iPSC differentiation toward HLCs represents a logical next step to investigate whether the distinctive hepatic phenotype observed clinically arises from intrinsic hepatocyte-specific mechanisms. In contrast to *in vivo* studies, where hepatic metabolism is influenced by adipose tissue, endocrine signals, dietary factors, and neural regulation, HLCs allow direct assessment of cell-autonomous metabolic pathways under controlled experimental conditions.

Several hepatic processes relevant to PWS could be investigated using patient-derived HLCs. These include *de novo* lipogenesis, mitochondrial β-oxidation, VLDL secretion, lipid droplet dynamics, and insulin responsiveness. HLCs would also provide an opportunity to evaluate the expression and function of hepatic proteins associated with steatosis in PWS, including CES1, FBP1, and QDPR, which have been identified through circulating proteomic analyses (57).

An additional advantage of iPSC-based approaches is the ability to compare patients with distinct genetic subtypes of PWS. HLCs derived from individuals with paternal deletions, maternal uniparental disomy, or imprinting defects could reveal whether hepatic phenotypes differ according to the underlying genetic mechanism. Although clinical studies suggest broadly similar rates of MASLD across the major genetic forms of PWS, patient-derived HLCs may reveal molecular differences that are not apparent from clinical observations alone (Graziano 65). Furthermore, recently developed isogenic stem cell models carrying engineered deletions within the 15q11-q13 region offer an opportunity to directly attribute hepatic phenotypes to the PWS locus while minimizing inter-individual genetic variability (82).

Such models may be particularly useful for investigating the contribution of SNORD116, which is increasingly recognized as a key regulator of metabolic pathways in PWS (35). While SNORD116 deficiency disrupts adipogenic networks involving PPARG, CEBPA, and PRDM16, its potential role in hepatocyte-specific pathways remains unknown. HLC models could therefore be used to determine whether SNORD116 influences PC metabolism, mitochondrial function, VLDL secretion, or lipid droplet accumulation, providing a mechanistic link between the molecular genetics of PWS and its paradoxical hepatic phenotype.

The usefulness of HLCs may even extend beyond disease modeling and toward therapeutic development. Recent studies have demonstrated stable correction of epigenetic defects at the Prader-Willi syndrome imprinting control region (PWS-ICR) in iPSC-derived neural models (83). Applying similar strategies during hepatic differentiation could enable rescue experiments that distinguish primary effects of imprinting defects from secondary metabolic consequences, thereby providing an experimental framework for evaluating potential therapeutic interventions.

### 3.2 Liver organoids in genetic hepatic diseases

While HLCs provide valuable information at the cellular level, liver organoids offer a more physiologically relevant platform that better reproduces the multicellular complexity and three-dimensional (3D) organization of liver tissue. Notably, organoids are self-organizing 3D structures derived from pluripotent or adult stem cells that recapitulate key architectural and functional features of the native organ. Compared with conventional monolayer cultures, liver organoids exhibit greater cellular maturity, preserve cell-cell interactions, and more closely mimic the hepatic microenvironment (84, 85).

Liver organoids can be generated from primary hepatocytes (86), adult liver stem cells (87), or iPSCs. Among these approaches, iPSC-derived organoids are particularly attractive for studying inherited diseases because they retain the patient’s genetic background while allowing incorporation of multiple hepatic cell types, including hepatocytes, stellate cells, cholangiocytes, endothelial cells, and macrophages (88). This multicellular composition enables investigation of complex processes such as inflammation, fibrosis, and metabolic dysfunction that cannot be adequately modeled in isolated hepatocyte cultures.

Recent advances have significantly improved the physiological relevance of liver organoids. Enhanced protocols now support prolonged culture, improved hepatocyte maturation, maintenance of bile acid signaling pathways, and the establishment of zonation-like characteristics that resemble periportal and pericentral regions of the liver (89). These developments are particularly relevant to metabolic liver disease because lipid metabolism, oxidative stress, and steatosis often display strong zonal heterogeneity.

In PWS research, liver organoids provide a unique opportunity to investigate the mechanisms underlying the apparent protection from MASLD despite severe obesity and hyperphagia. Patient-derived organoids could be challenged with free fatty acid loading to mimic lipotoxic conditions while independently modulating insulin signaling pathways. Such experiments would allow investigators to dissect the relative contribution of lipid overload and insulin resistance, two major drivers of steatotic liver disease that appear to be partially uncoupled in PWS (42).

Comparative studies between organoids derived from PWS patients and those generated from individuals with nonsyndromic obesity could further clarify whether the relative hepatic protection observed in PWS reflects intrinsic hepatocyte properties or arises from systemic factors outside the liver. If PWS-derived organoids exhibit reduced lipid accumulation under identical experimental conditions, this would support the existence of cell-autonomous protective mechanisms. Conversely, similar responses across groups would suggest that protection emerges from interactions involving adipose tissue, endocrine signaling, or central nervous system regulation.

The incorporation of hepatic stellate cells and macrophages into PWS organoid models may also help address an important unresolved question: whether the reduced susceptibility to fibrosis reported in PWS is driven by intrinsic hepatic mechanisms or by altered inflammatory signaling. Given that fibrosis progression depends on complex interactions among hepatocytes, immune cells, and stromal populations, multicellular organoids represent an ideal platform for studying these processes (90).

Despite their considerable promise, iPSC-derived liver organoids remain subject to several limitations, including incomplete maturation, coexistence of fetal and adult cellular characteristics, variability among differentiation protocols, and the potential accumulation of reprogramming-associated abnormalities (88, 91, 92). Nevertheless, their ability to reproduce patient-specific liver biology while supporting sophisticated genetic and functional manipulation makes them among the most promising platforms currently available for studying hepatic disease mechanisms.

Taken together, HLCs and liver organoids provide complementary systems for investigating hepatic involvement in PWS. HLCs offer a reductionist approach to dissect cell-autonomous metabolic pathways, whereas organoids enable interrogation of multicellular interactions and tissue-level responses. Their integration with patient-derived and isogenic stem cell models may ultimately clarify the mechanisms underlying the paradoxical hepatic phenotype of PWS and provide a foundation for the development of targeted therapeutic strategies.

**Figure 1:**
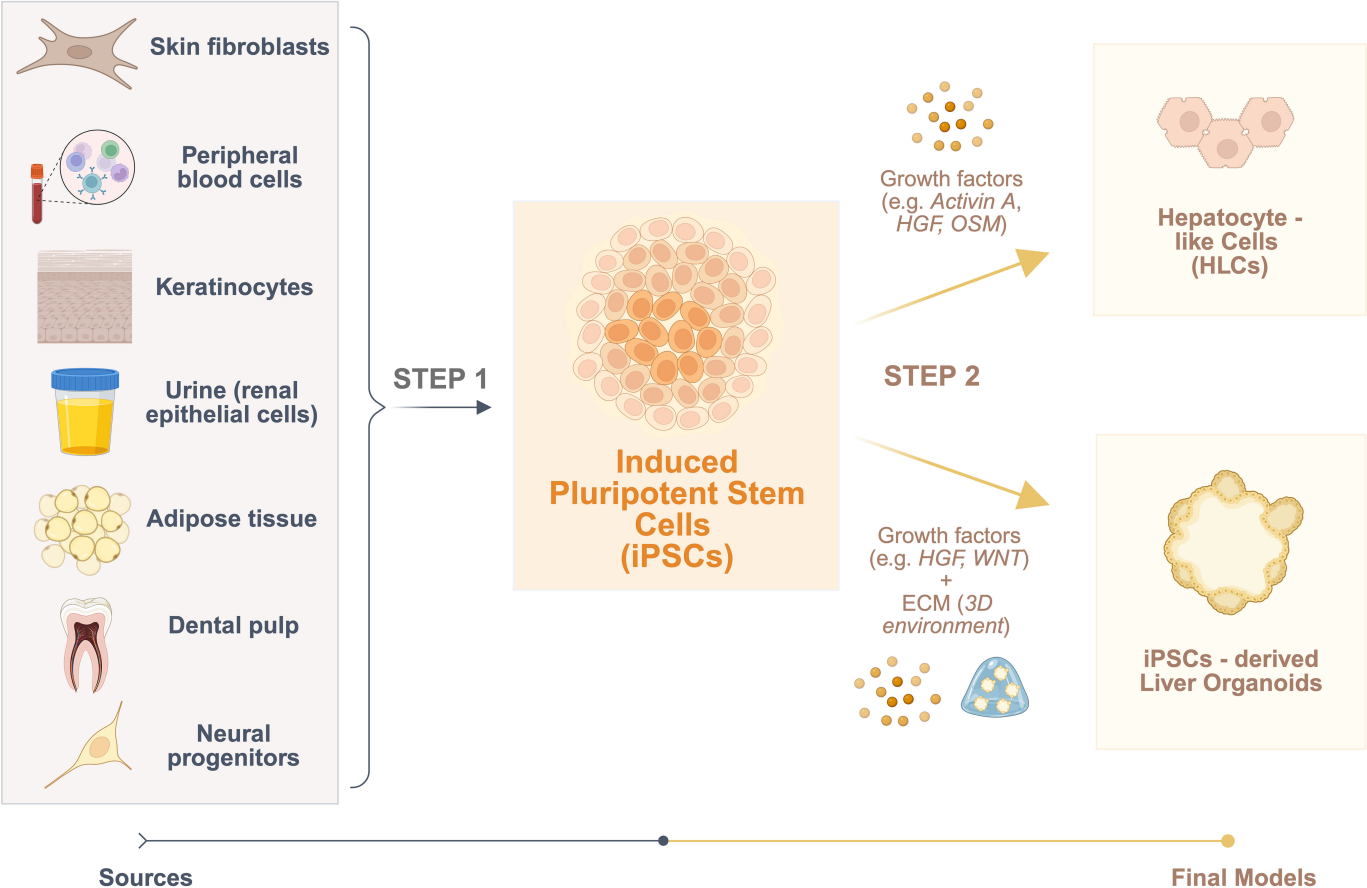
Generation of iPSCs from multiple somatic cell sources and differentiation into liver models. Schematic overview of iPSC generation from diverse human somatic cell sources, including skin fibroblasts, peripheral blood cells, keratinocytes, urinary epithelial cells, adipose tissue-derived cells, dental pulp cells, and neural progenitors. Following cell isolation and reprogramming (Step 1), iPSCs can be directed toward hepatic lineages (Step 2) through exposure to stage-specific growth factors and signaling cues, giving rise to 2D HLCs or 3D iPSC-derived liver organoids cultured within ECM-based environments. Created with BioRender.com. iPSC, induced pluripotent stem cell; HGF, hepatocyte growth factor; OSM, oncostatin M; HLC, hepatocyte-like cell; ECM, extracellular matrix.

**Figure 1** provides an overview of the diverse somatic cell sources that can be reprogrammed into iPSCs and of the downstream differentiation strategies used to generate two-dimensional (2D) and 3D hepatic models for disease modeling.

## 4 PWS and hepatic disease modeling

Human stem cell-based disease modeling, particularly using iPSCs combined with isogenic genome and epigenome editing, has emerged as a powerful strategy to dissect tissue-specific mechanisms underlying PWS phenotype. A key consideration in modeling PWS using iPSCs is whether epigenetic marks at the PWS-ICR are preserved during nuclear reprogramming. Due to extensive epigenetic remodeling during reprogramming, it has been argued that imprinting at chromosome 15q11-q13 may be vulnerable to erasure, potentially compromising iPSC-based models of PWS and related imprinting disorders (93). Although aberrant methylation has been reported at other imprinted loci following reprogramming, most studies indicate that methylation at the PWS-ICR is largely maintained, with control and PWS-derived iPSCs generally retaining the disease-specific methylation patterns present in their parental fibroblasts (93, 94).

The paradoxical combination of severe obesity with relative protection from insulin resistance and hepatic steatosis presents a major challenge for *in vivo* studies (95–97). This section reviews insights from iPSC-based metabolic models and epigenetic interventions, with emphasis on lipid metabolism and hepatic disease risk.

### 4.1 iPSCs and lipid metabolism

Clinical observations of preserved insulin sensitivity in PWS rely largely on systemic surrogate measures, which obscure tissue-specific metabolic defects. iPSC-based models provide a controlled platform to interrogate cell-autonomous phenotypes in adipocytes and hepatocytes. While early adipogenic commitment is largely preserved in PWS-derived adipocytes, defects emerge during terminal differentiation, particularly in pathways governing lipid trafficking, mitochondrial function, and insulin responsiveness (95).

Reduced expression of FABP4 and PPARG coactivator 1 alpha (PPARGC1A) is a consistent molecular feature, implicating impaired intracellular lipid flux and oxidative capacity in PWS iPSC-derived adipocytes (97). Dysregulated lipid droplet dynamics, including reduced perilipin 1 (PLIN1) expression and compensatory upregulation of lipolytic enzymes, further indicate inefficient lipid turnover rather than simple lipolytic failure (98–100). Functionally, PWS adipocytes exhibit increased lipid accumulation and impaired insulin-stimulated glucose uptake, revealing an intrinsic insulin resistance that is masked at the systemic level (95).

Isogenic genome-editing approaches strengthen causal inference by minimizing background variability. Megabase-scale deletions encompassing the 15q11–q13 region reproduce key metabolic phenotypes within a single genetic background, supporting a direct role for the PWS locus in adipocyte dysfunction (82). From a hepatic perspective, these adipocyte defects likely influence hepatic lipid flux, suggesting that apparent protection from MASLD arises from systemic compensation rather than intrinsically healthy adipose tissue.

**Figure 2:**
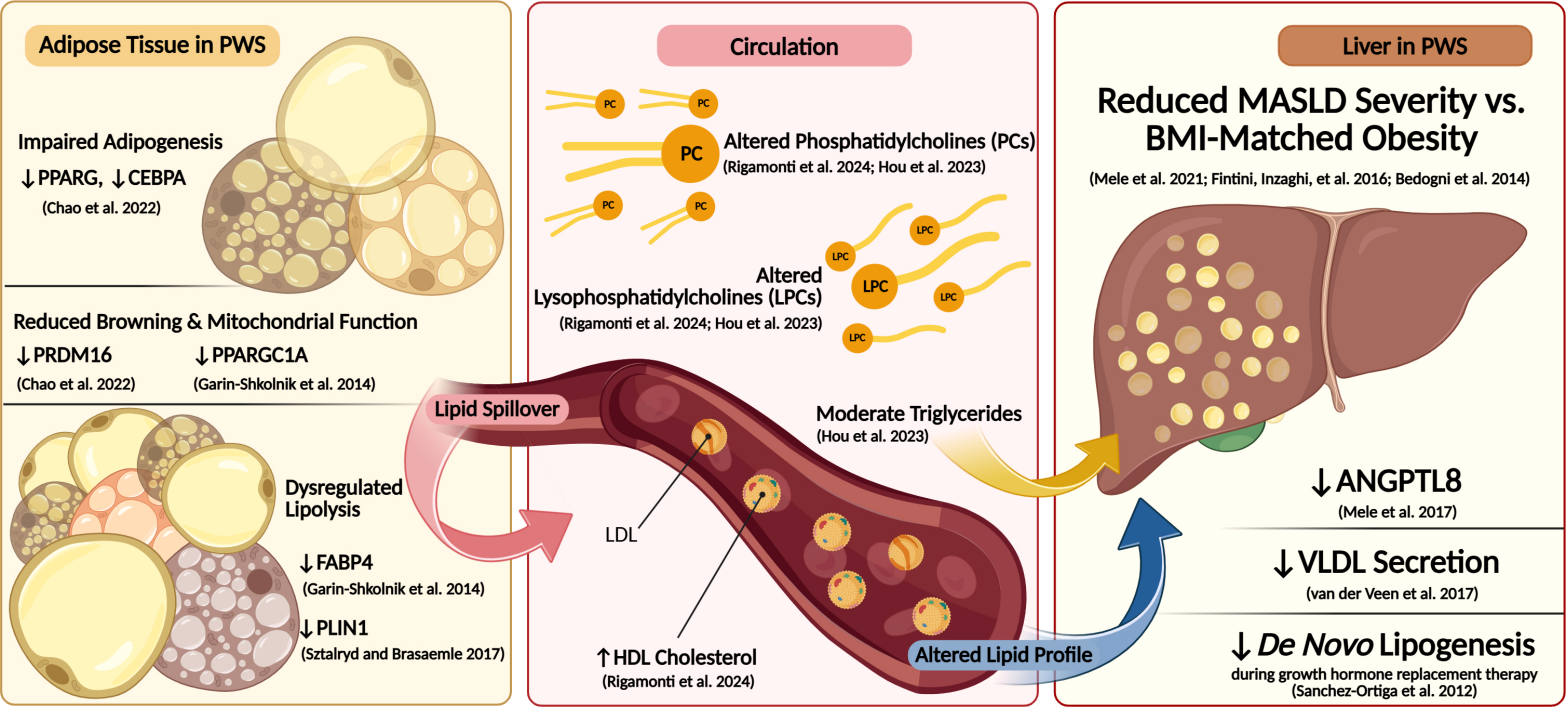
Hypothesized model of lipid metabolism and hepatic steatosis risk in Prader-Willi syndrome. The alterations in adipose tissue function, circulating lipid profiles, and hepatic metabolism in PWS are summarized from findings reported across multiple independent studies (35, 41, 42, 44, 55, 97–100). The relationships shown are conceptual and do not imply causality, as the temporal hierarchy and interdependence of these processes remain undefined. Created with BioRender.com. PPARG, peroxisome proliferator-activated receptor gamma; CEBPA, CCAAT enhancer binding protein alpha; PRDM16, PR/SET domain 16; PPARGC1A, PPARG coactivator 1 alpha; FABP4, fatty acid binding protein 4; PLIN1, perilipin 1; PC, phosphatidylcholine; LPC, lysophosphatidylcholine; VLDL, very-low-density lipoprotein; LDL, low-density lipoprotein; HDL, high-density lipoprotein; MASLD, metabolic dysfunction–associated steatotic liver disease; ANGPTL8, angiopoietin-like 8.

**Figure 2** summarizes reported alterations in adipose tissue function, circulating lipid profiles, and hepatic metabolism in PWS based on findings from multiple independent studies. Suggesting that qualitative remodeling of circulating lipid species and hepatokine signaling may contribute to a lower prevalence and severity of hepatic steatosis compared with nonsyndromic obesity. This framework highlights convergent metabolic features while underscoring the need for integrative studies to define causal pathways linking adipose dysfunction to hepatic disease risk.

## 5 From disease modeling to a potential therapeutic strategy

The transition from descriptive disease modeling toward mechanistic and therapeutic insight in PWS has been critically enabled by the development of advanced human stem cell-based systems. iPSCs, together with their derivatives, HLCs and organoids, provide an opportunity to investigate PWS pathophysiology in a human, patient-specific, and tissue-resolved manner. Unlike traditional animal models, these platforms preserve the complex genetic and epigenetic context of the PWS locus.

Given the epigenetic basis of PWS, disease pathology results from imprinting-mediated silencing of an intact maternal allele rather than irreversible gene loss. Targeted epigenome editing of the PWS-ICR has demonstrated that locus-specific demethylation is sufficient to restore expression of multiple PWS-associated genes in patient-derived iPSCs (83).

In the hepatic context, iPSC-derived HLCs and liver organoids allow the dissection of cell-autonomous metabolic phenotypes. These models enable direct investigation of lipid handling, mitochondrial function, and metabolic zonation under controlled conditions. Importantly, reactivated gene expression persists following differentiation into hypothalamic organoids, indicating stability across lineage commitment (83). Hierarchical demethylation extending beyond the PWS-ICR supports a model in which this region functions as a master regulator of locus-wide epigenetic state.

Complementary therapeutic strategies may target downstream metabolic regulators. ATP-sensitive potassium (K_ATP_) channels integrate cellular energy status with neuronal and metabolic function and represent a convergence point for appetite regulation, insulin secretion, and lipid metabolism. Pharmacological activation of K_ATP_ channels may ameliorate hyperphagia, improve lipid oxidation, and reduce hepatic lipid burden, offering a functional counterbalance to entrenched metabolic dysfunction (101).

Given that the development of PWS-specific iPSC-derived HLCs and liver organoids may have important translational implications beyond disease modeling, these platforms could be used to identify hepatic pathways that contribute to the distinctive metabolic phenotype of PWS and to evaluate candidate therapeutic targets involved in lipid metabolism, mitochondrial function, and insulin signaling. Furthermore, patient-derived models may support personalized medicine approaches by enabling comparison of treatment responses across different molecular subtypes of PWS, including paternal deletions and maternal uniparental disomy. Such systems could also facilitate preclinical testing of emerging epigenetic therapies aimed at restoring gene expression within the PWS locus. Ultimately, integrating patient-specific stem cell models with genome and epigenome editing technologies may provide a framework for linking molecular mechanisms to individualized therapeutic strategies in PWS.

Taken together, iPSC-derived HLCs and organoids bridge the gap between molecular epigenetic mechanisms and organism-level metabolic phenotypes in PWS. Their ability to capture patient-specific epigenetic regulation and tissue-specific metabolic dysfunction positions them as indispensable tools for advancing both mechanistic understanding and therapeutic development in PWS.
